# GL261 luciferase-expressing cells elicit an anti-tumor immune response: an evaluation of murine glioma models

**DOI:** 10.1038/s41598-020-67411-w

**Published:** 2020-07-03

**Authors:** Victoria E. Sanchez, John P. Lynes, Stuart Walbridge, Xiang Wang, Nancy A. Edwards, Anthony K. Nwankwo, Hannah P. Sur, Gifty A. Dominah, Arnold Obungu, Nicholas Adamstein, Pradeep K. Dagur, Dragan Maric, Jeeva Munasinghe, John D. Heiss, Edjah K. Nduom

**Affiliations:** 1 Surgical Neurology Branch, National Institute of Neurological Disorders and Stroke, National Institutes of Health, Bethesda, MD USA; 2 Flow Cytometry Core Facility, National Heart, Lung, and Blood Institute, Bethesda, MD USA; 3 Flow Cytometry Core Facility, National Institute of Neurological Disorders and Stroke, National Institutes of Health, Bethesda, MD USA; 4 Mouse Imaging Facility, National Institute of Neurological Disorders and Stroke, National Institutes of Health, Bethesda, MD USA; 5 Surgical Neurology Branch, National Institute of Neurological Disorders and Stroke, NIH, Room 3D-20, 10 Center Drive, Bethesda, MD 20892 USA

**Keywords:** CNS cancer, Immunotherapy, Neuroimmunology

## Abstract

Preclinical models that reliably recapitulate the immunosuppressive properties of human gliomas are essential to assess immune-based therapies. GL261 murine glioma cells are widely used as a syngeneic animal model of glioma, however, it has become common practice to transfect these cells with luciferase for fluorescent tumor tracking. The aim of this study was to compare the survival of mice injected with fluorescent or non-fluorescent GL261 cells and characterize the differences in their tumor microenvironment. Mice were intracranially implanted with GL261, GL261 Red-FLuc or GL261-Luc2 cells at varying doses. Cytokine profiles were evaluated by proteome microarray and Kaplan–Meier survival analysis was used to determine survival differences. Median survival for mice implanted with 5 × 10^4^ GL261 cells was 18 to 21 days. The GL261 Red-FLuc implanted mice cells did not reach median survival at any tumor dose. Mice injected with 3 × 10^5^ GL261-Luc2 cells reached median survival at 23 days. However, median survival was significantly prolonged to 37 days in mice implanted with 5 × 10^4^ GL261-Luc2 cells. Additionally, proteomic analyses revealed significantly elevated inflammatory cytokines in the supernatants of the GL261 Red-FLuc cells and GL261-Luc2 cells. Our data suggest that GL261 Red-FLuc and GL261-Luc2 murine models elicit an anti-tumor immune response by increasing pro-inflammatory modulators.

## Introduction

Glioblastoma (GBM) is the most common malignant primary brain tumor in adults^1^. Despite ongoing studies and numerous clinical trials, the prognosis remains poor^2^. There is an urgent need to provide these patients with new therapies, as the standard of care treatment has gone unchanged for more than a decade^3^. In light of the promising advances made in the area of immune therapy as a treatment for various solid cancers^4–7^, the field of neuro-oncology has embraced immunotherapies for gliomas as a promising area of preclinical and clinical investigation^8^. However, recent trials like the Phase III CheckMate-143^9^ and Phase III CheckMate-498^10^ show poor efficacy despite promising results from pre-clinical studies^11–15^. The lack of translation from preclinical to clinical studies illustrates the need for a more rigorous characterization of the pre-clinical models currently being used in the scientific community. The development of novel immune therapeutics is not possible without a reliable preclinical animal model that accurately mimics the complex immune landscape of GBM.

Of the various classes of preclinical mouse models, syngeneic models have been indispensable for evaluating immune therapies in GBM^16^. Syngeneic murine models are models that rely on allografts of immortalized cancer cells from the same mouse strain from which the model originates. This allows for the engraftment of tumors in the brain without immediate immune rejection. The most widely used syngeneic model of glioblastoma is the GL261 (mouse glioma 261) murine model^17^. However, instead of using the canonical model, luciferase-expressing cell lines like GL261-Luc2 and GL261 Red-FLuc are more commonly used in recently published preclinical studies ^13,18,19^.

In our study, we evaluated several pre-clinical glioma model systems to determine whether these models recapitulate the local and systemic immunosuppressive milieu of GBM patients. GL261 Red-Fluc, purchased from Perkin Elmer, and DNA plasmid-transfected GL261-Luc2 cells were insufficient to uniformly cause mortality in mice when injected at the same concentration as the parent GL261 cells. Our flow cytometry analysis showed significant differences in tumor infiltrating immune cells between mice injected with GL261 and GL261 Red-FLuc. Further, in vitro proteomic microarrays showed significantly elevated proinflammatory cytokines levels in both GL261-Luc2 and GL261 Red-FLuc cell lines in comparison to non-luciferase-expressing cells. These results collectively suggest a shift in the glioma tumor microenvironment to immunostimulatory when luciferase is expressed in GL261 cell lines.

## Results

### Implanted luciferase-expressing GL261 models prolonged survival in C57BL/6 mice

**Figure 1:**
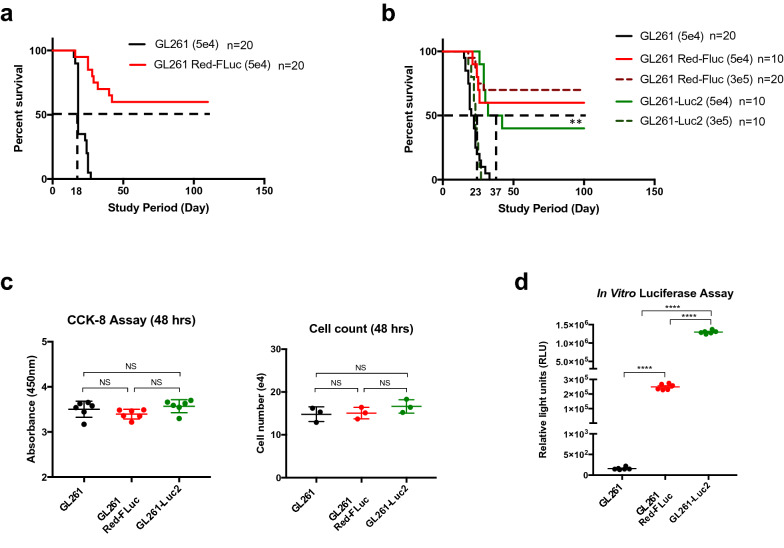
Outcomes for C57BL/6 mice intracranially injected with glioma cells. This figure represents an amalgamation of two separate identical experiments (n = 10 per group) which were performed under the same conditions. The combination resulted in a total of 20 mice per group implanted with 5 × 10^4^ cells. (**a**) Kaplan–Meier survival analysis demonstrate median survival for GL261 implanted mice is 18 days (n = 20). Alternatively, GL261 Red-FLuc implanted mice (n = 20) approached long-term survival with mice living over 100 days post tumor implantation (*P* < 0.0001, ****). (**b**) Displays median survival at increasing tumor doses. GL261 cells injected in mice at 5 × 10^4^ cells i.c. (n = 20) were used against the GL261 Red-FLuc implanted mice which did not demonstrate tumor burden at the 3 × 10^5^ (n = 20) or 5 × 10^4^ cells (n = 10) tumor doses. GL261-Luc2 cells reach median survival of 23 days for 3 × 10^5^ cells i.c. (n = 10) but exhibit significantly prolonged survival in mice implanted with 5 × 10^4^ cells i.c. at 37 days (n = 10) (*P* = 0.0011, **). (**c**) Measurements of proliferation rate for all three cell lines show no difference in in vitro doubling time. (**d**) Luciferase expression verified reporter gene expression in GL261 Red-FLuc and GL261-Luc2 cells (P < 0.0001, ****) All images in this figure were made using GraphPad Prism 8.3.0 (580) https://www.graphpad.com/scientific-software/prism/ and Microsoft PowerPoint Office 365 https://www.microsoft.com/enus/p/powerpoint/cfq7ttc0k7c6?activetab=pivot:overviewtab.

In order to validate controls for use in future preclinical work, we first implanted 5 × 10^4^ cells/5 μL of GL261 or GL261 Red-FLuc into the brains of C57BL/6 mice (Fig. 1a), expecting to find equivalent survival rates, as assessed by the Kaplan–Meier method. However, we found a significant difference in overall survival between these two groups (n = 20 per group, *P* < 0.0001). The GL261 implanted mice reached median survival at 18 days post-implantation with 100% of animals succumbing to disease after showing clear signs of clinical decline. Conversely, the GL261 Red-FLuc implanted mice did not reach median survival, and 60% of animals were described as long-term (≥ 100 days) survivors without neurological morbidity.

The significant differences found in overall survival prompted us to investigate if tumor dose or the type of luciferase expressed affect results found. We therefore evaluated an escalating tumor dose (Fig. 1b) of 3 × 10^5^ cells/5 μL of luciferase-expressing GL261 Red-FLuc or GL261-Luc2 cells against our starting dose of 5 × 10^4^ cells/5 μL. In this subsequent experiment, we found that mice implanted with 5 × 10^4^ cells of GL261 reached median survival at 21 days with 100% tumor penetrance (n = 20), similar to previous experiments. For GL261-Luc2 cells, the higher tumor dose of 3 × 10^5^ GL261-Luc2 cells (n = 10) approached the median survival of the GL261 mice dosed with 5 × 10^4^cells, and 100% symptomatic tumor penetrance. However, mice implanted with 5 × 10^4^ GL261-Luc2 cells (n = 10) demonstrated a significantly prolonged median survival of 37 days in comparison to GL261 implanted mice (*P* = 0.0011). Of note, 40% of animals implanted with 5 × 10^4^ GL261-Luc2 cells reached long term survival (> 100 days), without symptomatic tumor burden. In contrast, the mice implanted with either dose of GL261 Red-FLuc cells did not reach median survival, regardless of dose. Specifically, 60% of those implanted with 5 × 10^4^cells (n = 10) achieved long-term survival and 70% of those intracranially injected with a tumor dose of 3 × 10^5^cells (n = 20) were termed long-term survivors.

### GL261 and GL261 luciferase-expressing cells have similar proliferation rates in vitro

To explain the underlying differences found in overall survival, we next examined these cells for differences in cell growth and proliferation. First, we assessed in vitro proliferation of both cell lines via spectrophotometric CCK-8 assay and cell counting 48 h after seeding cells. We observed no differences for in vitro proliferation between the cell lines GL261 (3.503 ± 0.178, n = 6), GL261 Red-FLuc (3.395 ± 0.106, n = 6) and GL261-Luc2 (3.57 ± 0.144, n = 6) (Fig. 1c). In addition, we verified luciferase reporter gene and protein expression by ONE-Glo EX Luciferase Assay System (Fig. 1d). Here, we demonstrate that the original GL261 cells do not express luciferase, whereas the GL261 Red-FLuc and GL261-Luc2 cells are strongly positive for luciferase expression at (2.48 ± 0.08)10^5^ RLU (relative light unit) (n = 6) and (1.29 ± 0.2)10^6^ RLU (n = 6), respectively (P < 0.0001).

### GL261-red luciferase-expressing tumors recruit more pro-inflammatory cells to the brain microenvironment

#### Flow cytometry

**Figure 2:**
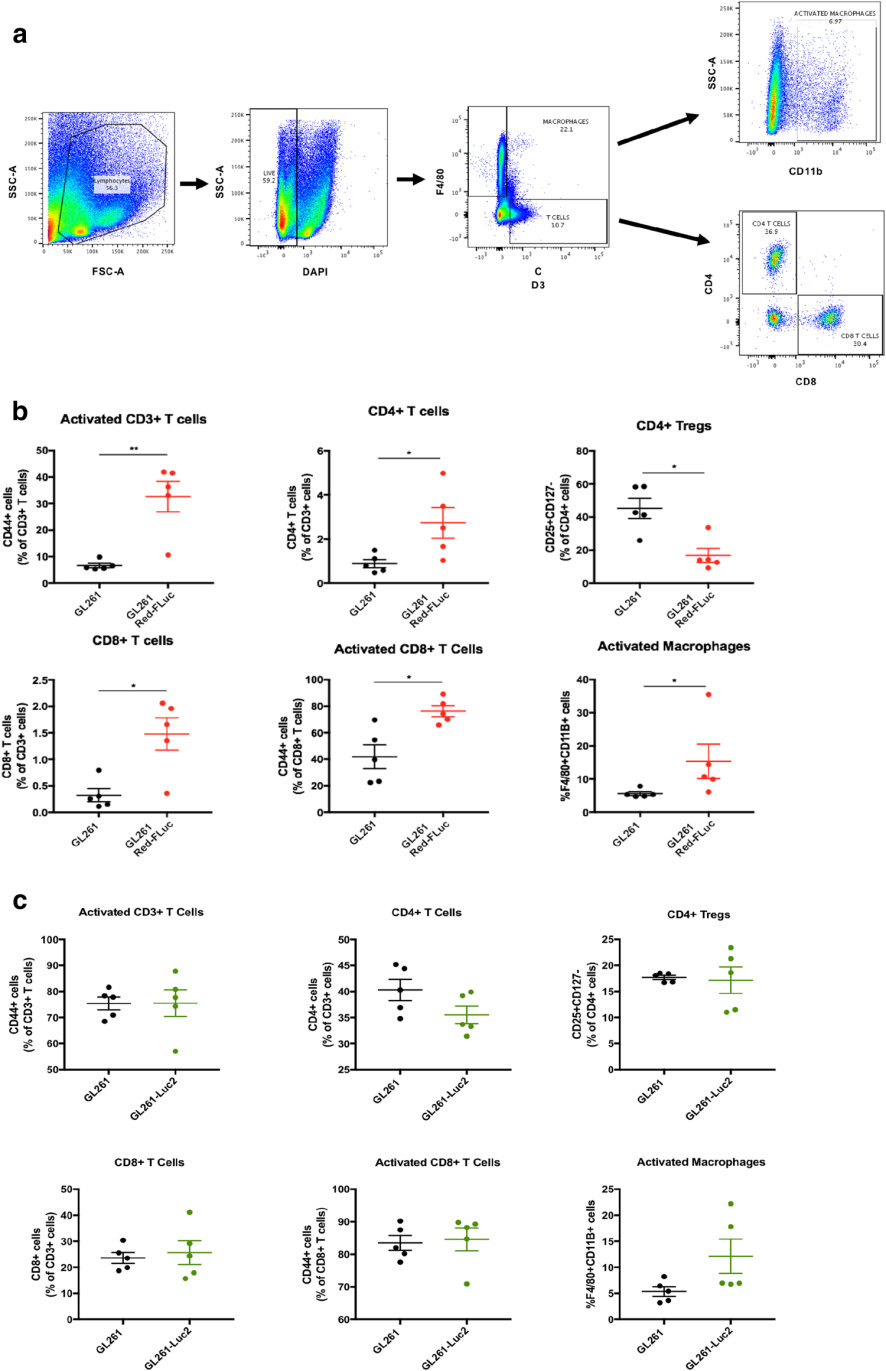
(**a**) Displays the various cell populations gated for F4/80 + macrophages and CD3^+^ T cells. CD3^+^ T-cell subsets were then fractioned by CD4 and CD8 positivity. (**b**) Shows the frequencies of immune cells observed in the GL261 and GL261 Red-FLuc implanted brains dissociated into a single cell suspension 10 days post-implantation. (**c**) Displays a panel of immune cell frequencies observed in GL261 and GL261-Luc2 implanted brains. Statistical significance was determined by Mann–Whitney U test with *P*-values of less than 0.05 considered statistically significant. All images in this figure were made using GraphPad Prism 8.3.0 (580) https://www.graphpad.com/scientific-software/prism/, Microsoft PowerPoint Office 365 https://www.microsoft.com/enus/p/powerpoint/cfq7ttc0k7c6?activetab=pivot:overviewtab and FlowJo v10.6.2 https://www.flowjo.com/solutions/flowjo/downloads.

Flow cytometry experiments were used to analyze the immune cell frequency in dissociated brain tissue cell suspensions from mice injected with 5 × 10^4^ cells/5 μL of GL261 or GL261 Red-FLuc tumor cell lines sacrificed 10 days following tumor implantation (n = 5 per group) (Fig. 2a). Day 10 was chosen to capture the immune infiltration into the GL261 tumors before growth caused significant morbidity but before luciferase-expressing tumors regressed. H + E from an additional cohort of mice that were implanted and sacrificed at day 10 demonstrated gross tumor formation in all 3 groups (Supplementary Fig. S1).

First, we compared the frequency of phenotypically sub-gated T-cells of mice injected with either GL261 or GL261 Red-FLuc (Fig. 2b). We observed a significant increase in the frequency of CD4^+^T cells of the GL261-injected mice (mean 0.8896%) compared to that of the GL261 Red-FLuc injected mice (mean 2.731%). Conversely, there was a significant decrease in CD4^+^Tregs (from mean 45.28% to 16.82%) in GL261 compared to the GL261 Red-FLuc. The CD8^+^T-cell population frequency showed a significant increase in the GL261 Red-FLuc (mean 1.478%) compared to GL261 (mean 0.324%). There was an increase in the frequency of CD8^+^Tregs in the GL261 group that did not reach significance (p = 0.08). When comparing macrophage populations, there was a significant increase in activated macrophages (F4/80^+^CD11b^+^cells) in GL261 Red-FLuc (mean 15.33%) compared to GL261 (mean 5.6%) tumors.

We repeated this flow cytometry analysis on mice injected with 5 × 10^4^ cells/5 μL of GL261 or GL261-Luc2 to compare immune cell frequencies between these two tumor cell lines (Fig. 2c). There was a slight observed increase in activated macrophages, however, there were no significant differences observed between brains of mice implanted with these two cell lines.

### Cytokine analysis shows significant differences in protein expression profiles

**Figure 3:**
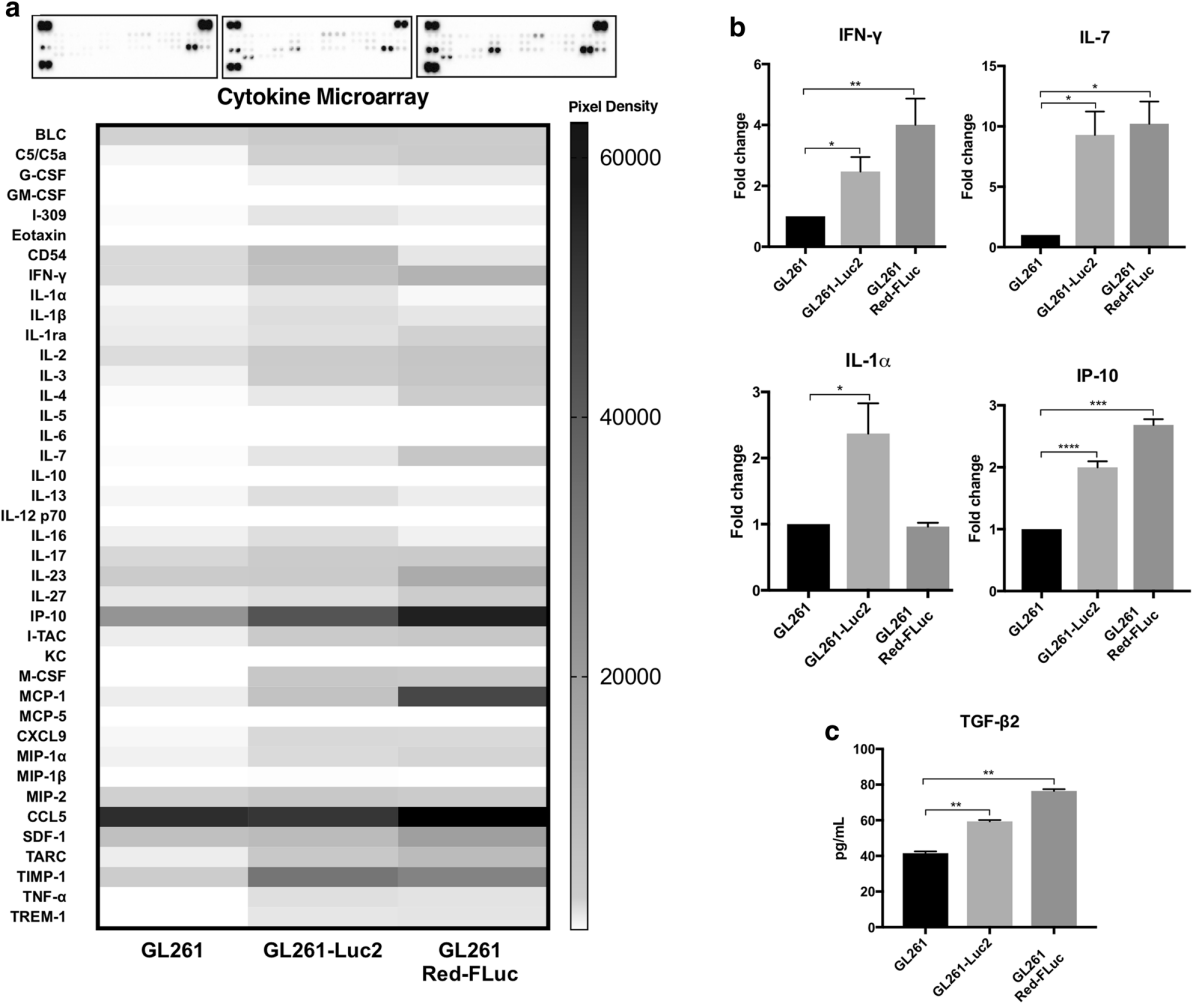
(**a**) Representative protein microarray images display differential cytokine profiles and raw pixel density for each group, respectively. (**b**) Relative fold change was determined and shown for cytokines of interest. The data are presented as means ± SEM, n = 3 for each group with (*P < 0.05, **P < 0.01, ***P < 0.001) by One-way ANOVA. Bar graphs show differential protein expression for selected cytokines of interest. (**c**) TGF-β2 ELISAs results show upregulated TGF-β2 expression measured in pg/mL for GL261 Red-FLuc and GL261-Luc2 compared to GL261 cells. All images in this figure were made using GraphPad Prism 8.3.0 (580) https://www.graphpad.com/scientific-software/prism/ and Microsoft PowerPoint Office 365 https://www.microsoft.com/enus/p/powerpoint/cfq7ttc0k7c6?activetab=pivot:overviewtab.

To further validate that the GL261 luciferase-expressing cell lines elicit an inflammatory response, we evaluated the secreted cytokine profiles for these cells in comparison to GL261 cells (Fig. 3). Overall, 15 of the 40 cytokines evaluated were significantly differentially expressed between the GL261 and GL261-RedFLuc (Fig. 3a). In addition, when comparing the expression profiles between the GL261 and GL261-Luc2, 12 cytokines were found to be significant in their expression. Some of these included proinflammatory cytokines such as IL-1alpha which was elevated greater than twofold in GL261-Luc2 (Fig. 3b). GL261 Red-FLuc and GL261-Luc2 cells also demonstrated an elevated expression in IFN-gamma, IL-7, and IFN-gamma-Induced Protein-10 (IP-10), among other immune stimulatory cytokines. Lastly, transforming growth factor-β2 (TGF-β2) expression was measured by ELISA of secreted cell supernatants (Fig. 3c). There was a significant upregulation in TGF-β2 expression in GL261 Red-FLuc (76.43 ± 1.024 pg/mL, n = 4) and GL261-Luc2 (59.38 ± 0.748 pg/mL, n = 4) compared to GL261 cells (41.55 ± 0.966 pg/mL, n = 4).

### MRI tumor assessment correlates with the onset of clinical symptoms and overall survival

**Figure 4:**
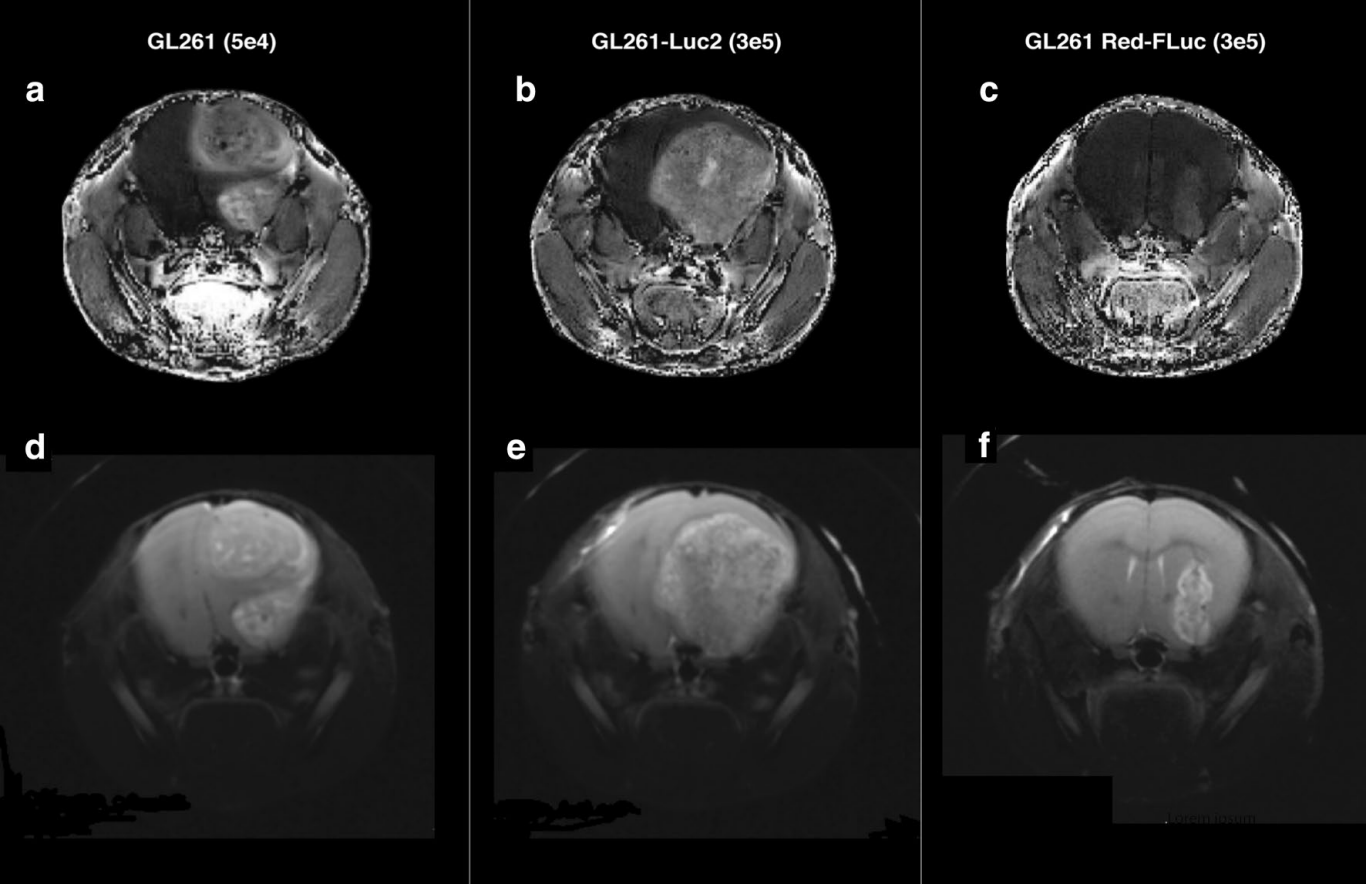
T1-weighted MRI images (top) showing contrast-enhancing lesions for all three groups (**a**) GL261, (**b**) GL261-Luc2, and (**c**) GL261 Red-FLuc implanted tumors at respective tumor dose. T2-weighted MRI (bottom) of (**d**) GL261, (**e**) GL261-Luc2, and (**f**) GL261 Red-FLuc tumors with tumor volumes correlating with onset of neurological deficits and symptoms All images in this figure were created using Microsoft PowerPoint Office 365 https://www.microsoft.com/enus/p/powerpoint/cfq7ttc0k7c6?activetab=pivot:overviewtab and Osirix DICOM Viewer https://www.osirix-viewer.com/.

Animals from all three groups were assessed by MRI at day 25 post intracranial injection. Asymptomatic mice implanted with 5 × 10^4^ GL261 cells, 3 × 10^5^ GL261-Luc2 cells, or 3 × 10^5^ GL261 Red-FLuc cells were evaluated for tumor growth (n = 1 per group). Images were processed and analyzed with MATLAB, and tumor volume was calculated using Osirix DICOM Viewer. The tumor volumes for the GL261 (Fig. 4a and d) and GL261-Luc2 cells (Fig. 4b and e) corresponded well with tumor dose at injection and survival time at 20.50 mm^3^ and 105.10 mm^3^, respectively. In the GL261 Red-FLuc implanted mouse (Fig. 4c and f), we found a tumor volume of only 2.48 mm^3^, which is also consistent with the prolonged survival times observed (Fig. 1a, b).

## Discussion

In order to improve immune therapy clinical trial results, we need to evaluate immune therapeutics in an immune-competent murine model system that accurately recapitulates several features of GBM, including immune suppression. While the field of neuro-oncology continues to make progress in the development of animal models of glioma, for expediency, we must make the best use of the available models for our ongoing translational work. Our study aims to underscore the importance of selecting an appropriate model for evaluating immune therapies, especially for an immunosuppressive neoplasm such as GBM.

Of the syngeneic cell lines available, GL261 is the most frequently studied. Recently, the GL261 Red-FLuc and GL261-Luc2 models have been used in several translational evaluations of immune therapy ^11–15,20–24^. These studies report median survivals varying greatly from 25 to 43 days. While Chae et al., Szatmari et al., and Plautz et al. describe the GL261 cells as rapidly fatal with mice reaching a moribund state within 21 days^20,25^ and never spontaneously regressing^26^, the same cannot be said for the luciferase-tagged cell lines as observed in our study. With our survival data, we demonstrated lifespan differences between mice injected with the tagged and untagged cell lines. These observations held true for animals injected with a higher tumor dose of 3 × 10^5^ GL261 Red-FLuc cells. Overall, the GL261 Red-FLuc cells are the most immunogenic of the three cell lines evaluated, with these tumors demonstrating poor engraftment with greater macrophage and T cell infiltrate at the site of tumor injection compared to all groups. In contrast, with the GL261-Luc2 cells, it appears that the number of glioma cells injected does correlate with survival. While we found that 3 × 10^5^ GL261-Luc2 cells are sufficient to induce mortality at the same rate as untagged GL261 cells, the same is not true for the lower tumor dose. Mice injected with 5 × 10^4^ GL261-Luc2 cells had a significantly prolonged survival, with 40% termed long-term survivors. Therefore, we postulate that increasing this glioma cell dose by as much as fivefold is sufficient for overwhelming the inherent immune response these cells would otherwise provoke at a reduced tumor burden. Our dose-dependent tumor growth experiment may explain why Clark et al. reports no difference in overall survival for GL261 and GL261-Luc cells when implanting a higher tumor dose of 3 × 10^5^ cells^27^. This finding led investigators to conclude that luciferase expression does not alter the immunologic properties of GL261 cells in 2014 despite finding significant differences in the transcriptional expression of Bimp-2, TGF-B2, IL-13, and CSF-3 (colony-stimulating factor 3), among others^27^.

In our study, intratumoral analyses via flow cytometry demonstrated significant differences in lymphocyte and macrophage infiltration between GL261 and GL261 Red-FLuc. Overall, the greater T-cell infiltration found in the GL261 Red-FLuc tumor microenvironment indicates a proinflammatory state^28^. Existing literature on T-cell immunity against tumors illustrates that T-cells are integral for targeting and eliminating tumor cells^29,30^. Glioblastoma, in particular, is known for evading immune surveillance^31–33^. It has been well established that gliomas gradually accumulate Tregs which blunt anti-tumor immune responses and thereby limit adaptive immunity^34^. CD4^+^FoxP3^+^ Tregs have been extensively studied, whereby an upregulation of this T-cell subset is shown to suppress T effector response and prompt T-cell apoptosis. In our study, we find an overall increase of Tregs in mice intracranially injected with GL261. The reduced frequency of FoxP3^+^ Treg cells in the GL261 Red-FLuc model further supports our hypothesis of a heightened immune response in these mice^30,35^. These observations suggest that the GL261 cells generate a tumor with an immunosuppressive microenvironment resembling GBM more so than GL261 Red-FLuc^16,36^.

Gliomas also escape immune surveillance through the secretion of inhibitory cytokines. Herein, we examine the cytokine expression of these cell lines in vitro. Chae et al. have previously reported cytokine expression differences between the GL261 and GL261 luciferase-expressing cells^20^. We detected, as they did, expression of monocyte chemoattractant protein 1 (MCP-1) across all cell lines, with elevated IL-4 (interleukin 4) in luciferase-expressing cells and minimal IL-6 expression across GL261 and GL261 luciferase-labeled cells. However, while they saw an elevation of CSF-1 in GL261 untagged cells, we observed an upregulation in the GL261 Red-FLuc and GL261-Luc2 cells^20^. The secreted cytokine profiles for these cells can vary due to the serum-free conditions used by Chae et al., unlike that used in our study. Overall, in concordance with the key differences we observed in IFN-gamma, IL-7, IL-1alpha, and TNF-alpha expression, there are previous reports of differential cytokine expression profiles observed for the GL261 and its luciferase-labeled cells. In addition, elevated TGF-β2 in GL261 luciferase-expressing cells is hypothesized to occur through a compensatory mechanism. It may resemble what was found by Ksendzovsky et al., whereby GL261 TGF-β1 secretion was found to be upregulated in the presence of T cell conditioned media and not elevated with Treg co-incubation^37^.

These findings have great relevance for translational research in neuro-oncology. Recent positive studies of checkpoint inhibition or checkpoint inhibition in combination with other immune therapeutics have nearly all used the GL261-Luc2 cell line as their immune-competent model^13,14,38,39^. Our data suggest that Luc2 expression may have primed these tumors for a response. This could mean that the results seen in these preclinical models would only translate to humans if the tumors treated were similarly immunogenic, which is not true for the pathologic state of GBMs.

While our study does not specifically elucidate the source of this immune response, we postulate that the presence of luciferase itself could be responsible. The expression of the luciferase gene has been shown to induce an immune response in wildtype mice and change the way subcutaneously injected melanoma cells responded to chemotherapy and targeted therapy^40^. Another study by Jeon et al. demonstrated that firefly luciferase also stimulated an immune response in immunocompetent BALB/c mice^41^. Similar concerns about the use of reporter-labeled tumor cells have been reported by Baklaushev et al. when they showed that orthotopic implanted Luc-expressing adenocarcinoma cells elicited an INF-gamma response in mice, protecting them from tumor engraftment^42^.

It is possible that the random luciferase insertion point disturbs the transcription of several key immune molecules, but this is not known. Further study is required to clarify these issues, but our aim is to inform those that the efficacy of immunotherapy in these models must be cautiously interpreted to thwart spurious results.

Murine models are widely used for studying immunotherapy in GBM. The preclinical studies of GBM tumor microenvironment are plagued with inconsistency and irreproducibility, thus impacting the rate at which therapies are developed. Having a standard murine model may address these issues. Our data suggest that the GL261 luciferase-labeled murine models create an unrepresentative microenvironment for tumor growth by increasing proinflammatory modulators. By identifying and utilizing pre-clinical models that approximate the tumor environment of GBM as accurately as possible, therapeutics targeting the human disease can be more effectively developed and tested. This may allow for better clinical trials in the future, with the bar for efficacy raised at the preclinical stage, possibly preventing the movement of ineffective therapeutics from the lab into our patients. Our work may lead to an improvement in preclinical studies for immune therapy for gliomas, as it provides a suggestion to increase the rigor of our investigations.

## Methods

### Cell culture and mice

The GL261 murine glioma cell line was obtained from the National Cancer Institute-Frederick Cancer Research Tumor Repository and maintained in RPMI-1640 medium with 10% fetal bovine serum (FBS). These cells were passaged (1:2) every 3 days to ensure logarithmic growth. GL261 Red-FLuc cells were obtained by Perkin Elmer Bioware Brite Cell Line (Waltham, MA). The GL261 Red-FLuc cells were grown in DMEM with 10% FBS and passaged (1:2) every 3 days. GL261 cells were transfected with DNA plasmid pGL4.51 (E1320), encoding luciferase reporter *luc2* using Promega FuGENE 6 transfection reagent. After establishment of a stable cell pool resistant to RPMI-1640 selection medium containing 200 ug/mL G418, single colonies were isolated by limited dilution. Reporter gene expression was verified by luciferase detection assay. All cell lines were maintained in a 37 °C humidified incubator with 5% CO_2_ and passaged to maintain 70% confluency. For tumor implantation, cells were trypsinized with 0.05% trypsin–EDTA, washed, and resuspended in phosphate buffered saline (PBS) at a final concentration of 5 × 10^4^ cells/5 μL or 3 × 10^5^ cells/5 μL. Six to eight-week-old C57BL/6 wild-type female mice were maintained at the National Institutes of Health’s Silvio O. Conte Animal Facility. The study was conducted in accordance with the NIH guidelines on the use of animals in research under approved Animal Study Protocol 1,404 by the Animal Care and Use Committee of NINDS.

### Stereotaxic intracranial tumor implantation

The mice were positioned in a David Kopf stereotaxic head frame and anesthesia mask (David Kopf Instruments, Tujunga, CA). Surgical anesthesia was maintained using 2% isoflurane mixed with oxygen. Following sanitation of the surgical area, a midline incision was made on the calvarium, extending from bregma to the lambda suture. The coordinates to the underlying right striatum target site were 2 mm posterior from bregma, 2 mm lateral from the coronal suture and 4 mm dorsal ventral from the exposed dura. Using a 10 μL gas-tight Hamilton syringe, a 5 μL volume containing glioma cells was stereotactically injected through a 1.2 mm burr hole in the calvarium into the underlying target site. Mice were randomized from different cages prior to tumor implantation. No more than 20 mice were intracranially injected in a single day to ensure the total time of the procedure did not exceed 2 h. Of the 20 mice, equal numbers of each group were injected. This was done to control for any variability that could occur from implanting tumors on different days. During the procedure, a mouse from each group was injected before another mouse from the same group. This controlled for the time differential between injections ensuring equivalence of the different groups. Glioma cells were prepared within 10–20 min prior to implantation to prevent a reduction in overall viability.

Animals were monitored daily to ensure humane endpoints with animals displaying a protruded skull, hunched posture, extreme lethargy, or weight loss as cause for euthanasia. Long-term survival was defined at 100 days post tumor implantation.

### Proliferation assay and luciferase reporter gene verification

To assess in vitro proliferation, GL261, GL261 Red-FLuc, and GL261-Luc2 cells were seeded in their optimal culture media at 10,000 cells/well in a 96-well plate suspended in 100 μL (n = 6 per group). The cells were pre-incubated for 48 h to allow for recovery and growth in a humidified atmosphere at 37 °C and 5% CO_2_. At 48 h, 10 μL of Cell Counting Kit-8 (CCK-8) reagent (Dojindo Molecular Technologies) was applied to each well and incubated for 2 h while a water-soluble formazan dye appears upon reduction by dehydrogenases in cells. The amount of formazan produced was directly proportional to the number of living cells and the colorimetric assay was then measured with a microplate reader with absorbance set to 450 nm. To validate CCK8 proliferation findings, we seeded cells at 6 × 10^4^ cells in a 6-well plate (n = 3 per group) and enabled growth over 48 h. At this time, we harvested cells with 0.05% trypsin and assessed viability and quantity with Trypan Blue.

To ensure reporter gene expression, we assayed the cells for luciferase expression using ONE-Glo EX Luciferase Assay System (E8110, Promega). Cells were seeded in a 96-well opaque white plate at 1 × 10^4^ cells/well in 100 μL of dispensed optimal culture media and allowed to grow and recover for 48 h. Then, cells were equilibrated to room temperature and ONE-Glo EX Reagent was applied at equal ratio to culture medium. Samples were placed on orbital shaker at 300 rpm for 3 min and assayed with luminometer (Synergy Neo2, BioTek).

### Flow cytometry of brain tumor infiltrative lymphocytes

Mice of a separate cohort from those undergoing survival assessments were intracranially injected with 5 × 10^4^ cells/5 μL of GL261, GL261 Red-FLuc, or GL261-Luc2 cells and sacrificed at day 10 (n = 5 per group). These mice were euthanized, and brains were immediately harvested and homogenized using gentleMACS Dissociator from Miltenyi Biotec (Gaithersburg, MD) with *Liberase* (Sigma Aldrich, St. Louis, MO) in RPMI. All brains harvested were similar in size, ranging from 0.38 to 0.40 g. After running the 37C_m_TDK_1 program on the dissociator, all brain tissue cell suspension samples were separated by density gradient using 1.03 g/μL and 1.095 g/μL density Percoll (Sigma Aldrich). Each sample was stained with 10ul of antibody and incubated for 15 min. The samples were then washed, resuspended in 100 μL of PBS, run on BDFACSAriaFUSION (BD, San Jose, CA) and analyzed for immune cell surface antigens.

### Flow cytometry analysis

UltraComp eBeads Compensation Beads (Thermo Fisher) were used for compensation control as were unstained mouse spleen cells. Cell suspension of brain tissue was stained with the cocktail of antibodies (see supplemental information for full list of antibodies) for cell surface antigens along with Live-Dead exclusion dye (Fixable Yellow viability stain, Invitrogen). A stained sample was run on BDFACSAriaFUSION (BD, San Jose, CA) equipped with 355, 407, 488, 532 and 633 nm LASER lines. Dead cells, debris and doublets were excluded in single-cell suspension by using FSC and SSC parameters and Live-dead dye combination. Further, live single cells were identified as Macrophage as F4/80^+^ CD3^-^ cells and activated macrophages as CD11b^+^F4/80^+^ cells. Tregs were identified as CD3^+^CD4^+^CD8^−^CD127^Lo^CD25^Hi^ among CD4 T cell fraction and CD3^+^CD4^−^CD8^+^CD127^Lo^CD25^Hi^ among CD8 T cell fraction. We also used CD44 antigen high expression in T cells subsets as a marker for activated T cells. Expression of CD152, CD279, and CD274 on Treg subsets were identified using FMO controls.

### Histology

Paraffin sectioning of fixed mouse brains was performed by Histoserv, Inc. (Germantown, MD 20874). Sections were serially cut at 5 μm thickness for each level until reaching the mid-area of the tumor localized to right, anterior frontal area of the mouse brain. Slides were dewaxed and stained with hematoxylin and eosin**.** Tumors were confirmed via H&E for animals sacrificed at the 10-day endpoint. All images were captured using Nikon Eclipse Ci light microscope.

### TGF-β2 ELISA

Murine glioma cell supernatants were applied to the ELISA plate pre-coated with monoclonal antibodies specific for mouse TGF-β2 (R&D Systems Quantikine ELISA). An enzyme-linked polyclonal antibody, the TGF-β2 conjugate, was then applied creating a quantitative sandwich enzyme immunoassay. Once the Substrate Solution and consecutive Stop Solution were added to each well, an enzyme reaction yielded a color change with varying intensities. The color intensity of each corresponded to a specific amount of TGF-β2 analyzed by Bio-Tek ELx800 UV–Vis microplate reader which calculated optical density. A standard curve was constructed by plotting mean absorbance for each lane of standard, and therefore allowed for the calculation of concentration in pg/mL of TGF-β2. Each sample was assayed in technical quadruplet (n = 4), and the experiment was performed three times with little variability between results.

### Proteome profiler mouse cytokine immunoassay

R&D Systems Proteome Profiler Mouse Cytokine Array Panel A consists of 40 capture antibodies spotted in duplicate on a nitrocellulose membrane. A cocktail of biotinylated detection antibodies was added to cell supernatants and these sample/antibody solutions were each added to and incubated on a separate membrane. Streptavidin-HRP and chemiluminescent reagents were added to the membranes and subsequently analyzed for light production at each spot via ChemiDoc MP Imaging System. Signal for each dot was calculated as integrated pixel density using Western Vision Quick Spots HLImage^++^ software. Three independent experiments were performed, all with similar results.

### Magnetic resonance imaging

All studies were performed in compliance with the guide for the care and use of Laboratory Animal resources (1996), National Research Council, and approved by the NINDS Animal Care and Use Committee. Mice were anesthetized with 1.5% isoflurane/air mixture and placed prone in a stereotaxic holder. The body core temperature was maintained at 37 °C with a heated circulating water jacket. Respiration and temperature were monitored throughout the experiment. The mouse head was mounted and centered in an 85 mm i.d. transmit and 4 receiver array radio frequency (rf) coil ensemble. The MRI experiments were performed on a horizontal bore 9.4 T scanner operating on a Bruker Avance platform (Bruker Biospin Inc., Bellerica, MA). In order to ensure that the head experienced the best magnetic homogeneity, performance of the transmit and receive rf coils were optimized with respect to the mouse head. Three mutually perpendicular scout images were acquired through the brain to help localize slice positions in subsequent scans. Anatomical delineation of the tumor region was achieved by acquiring T2 weighted axial slices through the entire brain using RARE (Rapid Acquisition with Relaxation Enhancement) sequence (Slice thickness [ST] = 1 mm, Field-of-view [FOV] = 19.2 mm and matrix size = 192 × 192, in-plane resolution of 100 μm, Effective echo time [TE] = 40 ms, repetition time [TR] = 3,000 ms, number of averages [NA] = 4), and RARE Factor = 8, Total Scan Time ~ 5mins.). Tumor volume was calculated using Osirix and images were processed with MATLAB at the NIH Mouse Imaging Facility.

### Statistical analysis

Please see supplemental information for full statistical analysis.

## Supplementary information

Supplementary file1 (PDF 293 kb)

## Data Availability

Authors declare that all data supporting the findings of this study are available within the article or its supplementary file, and available from the corresponding author upon request.
