# Author Correction: GL261 luciferase-expressing cells elicit an anti-tumor immune response: an evaluation of murine glioma models

**DOI:** 10.1038/s41598-022-09883-6

**Published:** 2022-04-04

**Authors:** Victoria E. Sanchez, John P. Lynes, Stuart Walbridge, Xiang Wang, Nancy A. Edwards, Anthony K. Nwankwo, Hannah P. Sur, Gifty A. Dominah, Arnold Obungu, Nicholas Adamstein, Pradeep K. Dagur, Dragan Maric, Jeeva Munasinghe, John D. Heiss, Edjah K. Nduom

**Affiliations:** 1 Surgical Neurology Branch, National Institute of Neurological Disorders and Stroke, National Institutes of Health, Bethesda, MD USA; 2 Flow Cytometry Core Facility, National Heart, Lung, and Blood Institute, Bethesda, MD USA; 3 Flow Cytometry Core Facility, National Institute of Neurological Disorders and Stroke, National Institutes of Health, Bethesda, MD USA; 4 Mouse Imaging Facility, National Institute of Neurological Disorders and Stroke, National Institutes of Health, Bethesda, MD USA; 5 Surgical Neurology Branch, National Institute of Neurological Disorders and Stroke, NIH, Room 3D‑20, 10 Center Drive, Bethesda, MD 20892 USA

Correction to: *Scientific Reports* 10.1038/s41598-020-67411-w, published online 03 July 2020

The original version of this Article contained an error in the Abstract.

“Preclinical models that reliably recapitulate the immunosuppressive properties of human gliomas are essential to assess immune-based therapies. GL261 murine glioma cells are widely used as a syngeneic animal model of glioma, however, it has become common practice to transfect these cells with luciferase for fluorescent tumor tracking. The aim of this study was to compare the survival of mice injected with fluorescent or non-fluorescent GL261 cells and characterize the differences in their tumor microenvironment.”

now reads:

“Preclinical models that reliably recapitulate the immunosuppressive properties of human gliomas are essential to assess immune-based therapies. GL261 murine glioma cells are widely used as a syngeneic animal model of glioma, however, it has become common practice to transfect these cells with luciferase for bioluminescent tumor tracking. The aim of this study was to compare the survival of mice injected with bioluminescent or non-bioluminescent GL261 cells and characterize the differences in their tumor microenvironment.”

The original Article has been corrected.

