# Supplementary material for: GL261 luciferase-expressing cells elicit an anti-tumor immune response: an evaluation of murine glioma models
Source: Sci Rep. 2020 Jul 3;10:11003. doi: 10.1038/s41598-020-67411-w (PMC7335060; doi:10.1038/s41598-020-67411-w)
Supplement: Supplementary file 1 — Supplementary file1 (PDF 293 kb) [file 41598_2020_67411_MOESM1_ESM.pdf]

GL261 luciferase-expressing cells elicit an anti-tumor immune response: an evaluation of murine glioma models

Victoria E. Sanchez[1], BS, John P. Lynes[1], MD, Stuart Walbridge[1], BS, Xiang Wang[1], MS, Nancy A. Edwards[1], BA, Anthony K. Nwankwo[1], BS, Hannah P. Sur[1], BS, Gifty A. Dominah[1], BA, Arnold Obungu[1], BS, Nicholas Adamstein[1], BA, Pradeep K. Dagur[2], PhD, Dragan Maric[3], PhD, Jeeva Munasinghe[4], PhD, John D. Heiss[1], MD, Edjah K. Nduom*[1], MD

[1]Surgical Neurology Branch, National Institute of Neurological Disorders and Stroke, National Institutes of Health, Bethesda, MD, USA

[2]Flow Cytometry Core Facility, National Heart, Lung, and Blood Institute, Bethesda, MD, USA

[3]Flow Cytometry Core Facility, National Institute of Neurological Disorders and Stroke, National Institutes of Health, Bethesda, MD, USA

[4]Mouse Imaging Facility, National Institute of Neurological Disorders and Stroke, National Institutes of health, Bethesda, MD, USA

Corresponding Author:

*Edjah K. Nduom, M.D.

Surgical Neurology Branch

National Institute of Neurological Disorders and Stroke, NIH

Room 3D-20

10 Center Drive

Bethesda, MD 20892

Phone: 301-480-0284

Fax: 301-402-0380

Email: edjah.nduom@nih.gov

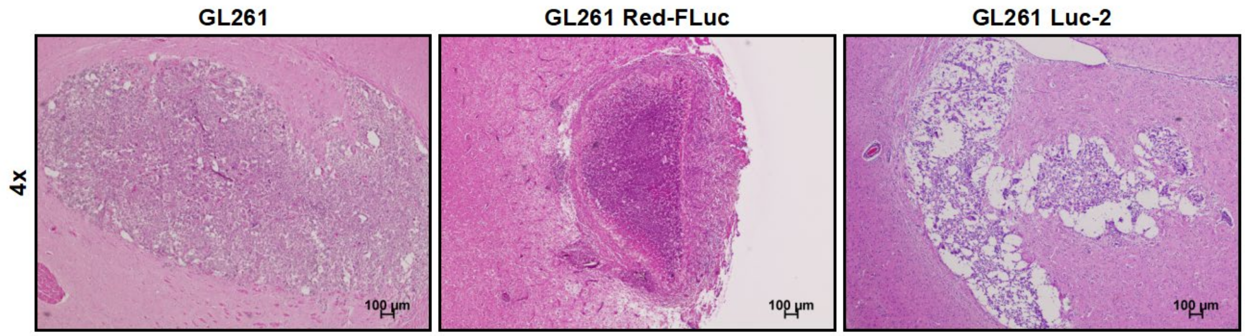

Supplementary Fig. 1 Representative H&E stained brains of mice sacrificed at the 10-day endpoint for GL261, GL261-RedFluc, and GL261-Luc2 implanted mice. Mice of this cohort were used for flow cytometry experiments and show sites of tumor implantation for each group, respectively.

<u>Supplemental Information</u>

Antibodies used for flow cytometry

Anti-mouse CD152 BV421 (106312, Biolegend, 1 µg/10^6 cells), Anti-mouse CD4 BV605 (563151, BD, 0.25 µg/10^6 cells), Anti-mouse CD8α BV650 (563234, BD, 0.125 µg/10^6 cells), Anti-mouse CD44 FITC (561859, BD, 0.06 µg/10^6 cells), Anti-mouse CD127 PerCP/Cy5.5 (135022, Biolegend, 0.25 µg/10^6 cells), Anti-mouse CD279 PE (551892, BD, 2 µg/10^6 cells), Anti-mouse CD11b PE/Cy5 (101210, Biolegend, 0.25 µg/10^6 cells), Anti-mouse CD25 PE-Cy7 (552880, BD, 0.25 µg/10^6 cells), Anti-mouse CD274 APC (564715, BD, 0.125 µg/10^6 cells), Anti-mouse F4/80 APC/Cy7 (123118, Biolegend, 1 µg/10^6 cells), Anti-mouse CD3e BUV395 (563565, BD, 0.25 µg/10^6 cells).

Statistical analysis

To assess survival differences observed between mice injected with glioma cells, distributions of overall survival were estimated using Kaplan-Meier method. A log-rank (Mantel-Cox) test was used to determine *P*-values. All groups were compared to the GL261 control. Animal deaths not related to tumor growth or severity of neurological deficit were excluded. Long term survival was set at 100 post injection. A threshold of $P<0.05$ was used to determine statistical significance for each experiment (*P<0.05, **P<0.01, ***P<0.001, ****P<0.0001). Unless otherwise stated, data were analyzed with GraphPad Prism 6 (GraphPad Software, Inc., San Diego, CA). All graphs indicated with mean and standard error of the mean (SEM).
